# AI-guided epitope engineering of a SARS-CoV-2 spike antigen for broad sarbecovirus neutralization

**DOI:** 10.3389/fimmu.2026.1919664

**Published:** 2026-09-18

**Authors:** Alexandru Odainic, Ioannis Vardaxis, Matheus Ferraz, Katrin E Wiese, Lisette A H M Cornelissen, Akhilesh Sharma, Richard Stratford, Trevor Clancy, Kaïdre Bendjama

**Affiliations:** 1 NEC Bio B.V., Amsterdam, Netherlands; 2 Wageningen Bioveterinary Research, Wageningen University and Research, Lelystad, Netherlands; 3 Department of Vaccine Bioinformatics, Institute of Tropical Medicine, Nagasaki University, Nagasaki, Japan

**Keywords:** artificial intelligence, broadly protective, coronavirus, immunoinformatics, vaccine

## Abstract

The rapid evolution of SARS-CoV-2 and the ongoing risk of zoonotic spillover highlight the need for vaccines that provide broad protection beyond strain-specific immunity. Here, we present a structure-guided, AI-enabled strategy for rational antigen design that enhances cross-reactive B-cell epitope recognition across betacoronaviruses. By integrating comparative sequence analysis with conformational epitope prediction, we identified conserved epitope hotspots within the Spike receptor-binding domain and introduced targeted motif-level substitutions into a SARS-CoV-2 BA.2 backbone. Engineered Spike immunogens, delivered as mRNA-lipid nanoparticle vaccines, elicited robust antibody responses in mice and demonstrated broad neutralizing activity against antigenically diverse SARS-CoV-2 variants, including BA.5 and XBB.1.5, as well as zoonotic sarbecoviruses. Selected designs reached neutralisation breadth comparable to an Omicron-adapted clinical benchmark for shared SARS-CoV-2 antigens, while extending neutralising activity to divergent zoonotic sarbecoviruses. These findings show that minimal, structure-guided epitope remodeling can enhance the presentation of conserved epitopes and improve antibody breadth, providing a generalizable framework for designing vaccines resilient to viral evolution and future pandemic threats.

## 1 Introduction

The global dissemination of SARS-CoV-2 underscored the threat posed by highly transmissible viruses originating from zoonotic spillovers (1, 2). The subsequent unprecedented effort to rapidly deliver COVID-19 vaccines (3, 4) was a landmark achievement, demonstrating the power of platform technologies such as mRNA and viral vectors (5–7). Yet, as the pandemic progressed, strain-specific vaccines proved vulnerable to viral evolution, with new variants reducing their effectiveness and necessitating updates (8, 9).

As SARS-CoV-2 spread globally, it accumulated mutations that repeatedly altered the antigenic repertoire of Spike protein. Successive waves of variants revealed how selective pressure imposed by pre-existing immunity, together with selection for enhanced viral fitness such as improved infectivity and transmissibility, can contribute to immune escape through remodeling of the target antigen surface (10). A central reason this cycle persists is that anti Spike elicited antibodies predominantly target the immunodominant but hypervariable surfaces of the Spike RBD. This region is under strong positive selective pressure, anchoring the humoral response to the antigenic configuration present at immunisation and rendering it inherently strain specific (11, 12). As a result, even after multiple exposures through vaccination, infection, or both, cross-neutralizing activity narrows as variants diverge, leaving populations repeatedly vulnerable to antigenically drifted strains (13, 14). This vulnerability extends beyond circulating variants. The sarbecovirus reservoir in wildlife harbours substantial antigenic diversity, and spillover of divergent strains would likely escape the strain specific immunity conferred by current vaccines entirely (15, 16).

This rapid evolution of SARS-CoV-2 has further highlighted the limitations of traditional vaccine design. Neutralizing antibodies elicited by current vaccines predominantly target the Spike receptor-binding domain (RBD), a region that accumulates mutations at a high rate (12). As new variants emerge, vaccine effectiveness declines, prompting recurrent updates to maintain protection (17). A comparable issue is observed in the case of influenza, where annual vaccine updates are necessitated due to antigenic drift. Strain selection must be performed several months prior to immunization campaigns, and the continual evolution of viral antigens frequently results in only moderate levels of protection (18, 19).

These examples highlight that a key goal in vaccinology is the engineering of antigens ensuring broad protection against various viral strains and species.

To achieve broad-spectrum efficacy, several complementary strategies have been explored. One strategy leverages multivalent formulations, such as the licensed bivalent or trivalent mRNA-1273.222 and BNT162b2 vaccines, to broaden antigen coverage (20, 21). Another approach consists of fusing antigenic epitopes from different variants, exemplified by the Delta BA.2 tandem-fusion heterodimer mRNA vaccine (22). It has also been investigated the targeting of conserved viral sequences to elicit responses less sensitive to antigenic drift (23). These strategies have yielded encouraging early results, demonstrating their potential to elicit broad-spectrum neutralizing antibodies through vaccination. Despite their promise, such approaches remain fundamentally reactive to the onset of new variants and therefore offer limited protection against future emerging SARS-Cov-2 strains (24).

Betacoronaviruses, as a group, pose additional concerns for global health security (15). Beyond SARS-CoV-2, this genus includes SARS-CoV, MERS-CoV, and several endemic human coronaviruses, all of which share ecological reservoirs in bats and other wildlife species (16). Their high recombination rates, broad host ranges, and increasingly frequent spillover events sustain the continual risk of future emergent lineages with pandemic potential (25, 26). The recurrent emergence of phylogenetically diverse coronaviruses over the past two decades suggests that SARS-CoV-2 is not the sole coronavirus threat (25, 26). Although there are conserved structural motifs within the Spike protein, specifically in the RBD, these regions are often immunologically subdominant, overshadowed during natural infection by the hypervariable regions that elicit strong but strain-specific antibody response (27, 28).

In this work, we present an epitope-focused engineering strategy to generate broadly protective sarbecovirus immunogens, derived from a betacoronavirus wide conserved epitope mapping framework. In our previous work (29), we described a bioinformatic pipeline designed to systematically identify B-cell epitopes within viral proteins. This pipeline integrates multiple sequence alignment with structural modeling to map both linear and conformational B-cell epitope candidates across diverse viral species. By combining sequence conservation analysis and three-dimensional epitope prediction, the approach enables the pinpointing of immunologically relevant regions that are most likely to elicit cross-reactive antibody responses.

Using this this AI framework, we introduced rational mutations into the Spike RBD sequence to enhance the presentation of conserved, cross-reactive epitopes. These engineered immunogens were then used to construct LNP-mRNA vaccines and evaluated experimentally by assessing neutralizing antibody responses across a panel of sarbecoviruses, including SARS-CoV-2 variants, SARS-CoV, and zoonotic sarbecoviruses. Our results show that the modified Spike antigens elicited broader and more cross-neutralizing antibody responses than the wild-type Spike protein.

These findings exemplify the strong potential of AI for the rational engineering of antigens which overcome the challenges posed by viral evolution and immune escape. This work establishes a framework for designing vaccine antigens with enhanced cross-protective capacity, with implications for preparedness against both current and future viral pandemic threats.

## 2 Results

### 2.1 Conserved RBD epitope mapping across betacoronaviruses

To identify conserved and cross-reactive B-cell epitope hotspots across betacoronaviruses as a foundation for rational immunogen mutagenesis and design, we first performed a comparative analysis of Spike RBDs spanning across the major betacoronavirus subgenera. We selected a representative group of 304 Spike protein sequences from different species and performed a multiple sequence alignment using BLOSUM62 substitution matrix to establish a shared residue-coordinate system for epitope mapping. This alignment is used only to place epitope candidates at homologous positions across strains and does not itself enter the epitope prediction, which evaluates the three-dimensional structural context of each mapped position. Out of the total, 290 sequences could be aligned within the RBD and retained for downstream analysis, enabling direct positional correspondence of epitope candidates across species. For each of the 290 aligned RBDs, B-cell epitope probabilities were computed using our previously described positional permutation vector (PPV) framework (29). Briefly, a PPV is a binary vector of length equal to the antigen sequence, in which each position denotes the status of the corresponding amino acid residue with respect to the epitope. In this representation, a value of 0 indicates an amino acid residue that is contained within the epitope but does not constitute an antibody contact site, whereas a value of 1 indicates an epitope residue that directly forms a contact point with the antibody. This formalism preserves the positional arrangement of functionally distinct epitope residues along the protein sequence and provides a compact representation for modeling epitope architecture as a global sequence pattern. PPVs capture local 3D structural context and sequence environment of the Spike RBD (**Figure 1**). Conformational B-cell epitope predictions were first generated within the SARS-CoV-2 RBD and then mapped onto the corresponding aligned regions of all other betacoronavirus RBDs (**Figures 2A–F**). Each conformational B-cell epitope constitutes a unique candidate among all betacoronavirus subgenera. For each epitope, conservation breadth was quantified by enumerating the species in which the predicted epitope probability surpassed the 0.9 threshold [selected based on internal benchmarking within the BCE-Hunt framework; see Methods (29)]. Using this criterion, epitope candidates were most frequently observed within the Sarbecovirus subgenus (**Figure 2A**), where 221 out of 224 species exhibited at least one conformational epitope exceeding the probability threshold. In contrast, fewer species met this criterion in the Embecovirus (21 of 30) (**Figure 2B**), Merbecovirus (9 of 20) (**Figure 2C**), Nobecovirus (5 of 7) (**Figure 2D**), Hibecovirus (1 of 1) (**Figure 2E**), and unclassified betacoronaviruses (4 of 8) (**Figure 2F**). The higher counts observed for Sarbecoviruses reflect both their greater representation in the dataset and the use of the SARS-CoV-2 RBD as the reference coordinate framework (**Figure 2G**). Specifically, epitope candidates (PPV) were defined on the SARS-CoV-2 RBD sequence using its residue coordinates and mapped onto other betacoronavirus RBDs through multiple sequence alignment, assigning epitope probabilities to homologous positions. As a result, epitope detection is biased toward motifs that are conserved in sequence and structural context relative to SARS-CoV-2, thereby increasing possible conservation within the Sarbecovirus subgenus. Together, this analysis revealed discrete coordinates within the RBD with high predicted epitope conservation across betacoronaviruses, providing a rational basis for the selecting candidate sites for antigen engineering by mutagenesis *in silico.*

**Figure 1:**
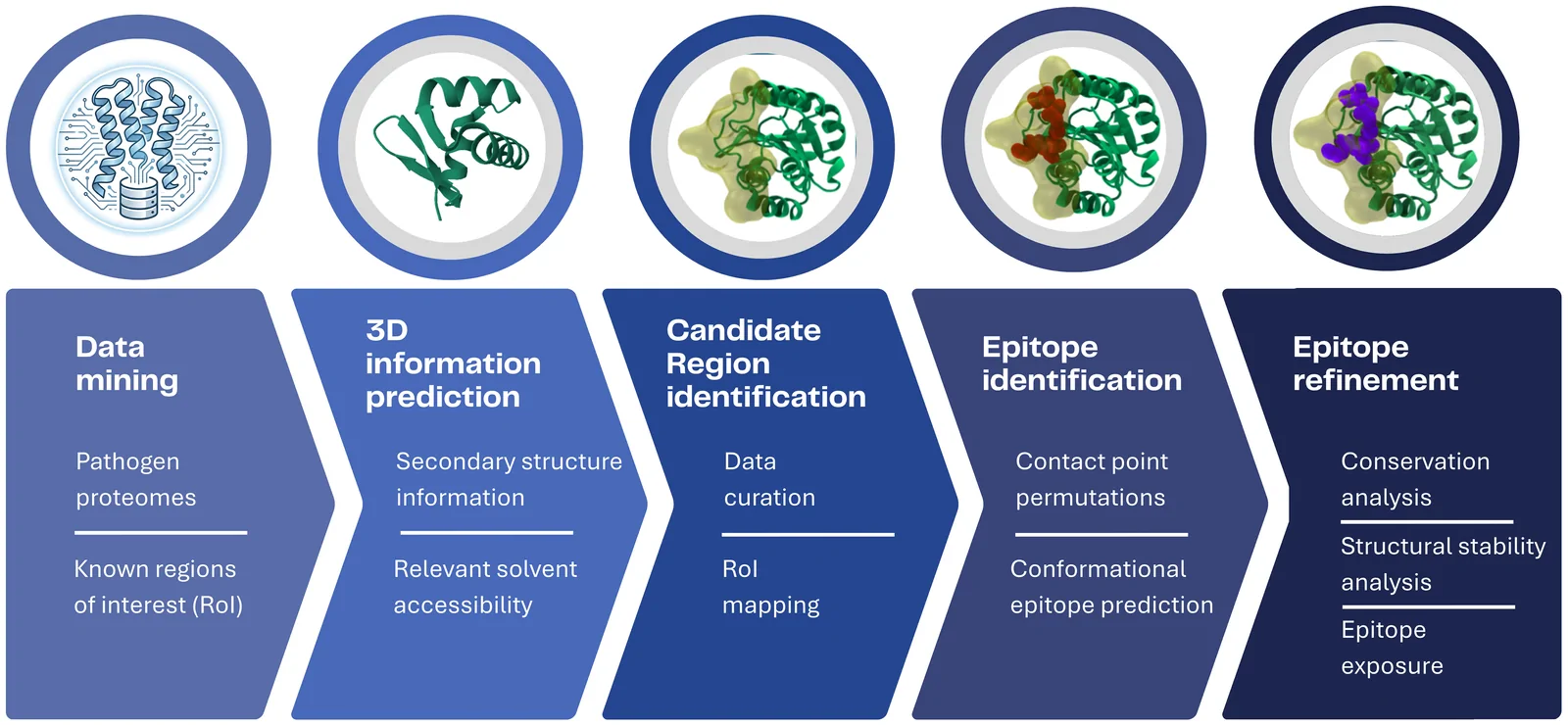
Structure guided pipeline for conformational B cell epitope discovery and optimization. Schematic overview of the computational pipeline. Pathogen genomic sequences were curated from public databases, and regions of interest were defined based on areas known to be targeted by neutralizing antibodies. Structural features required for conformational B cell epitope prediction, including secondary structure and relative solvent accessibility, were derived from three-dimensional modeling. Candidate regions were aligned to establish positional correspondence across strains. Conformational epitopes were predicted using a contact point permutation framework and subsequently evaluated based on structural stability, sequence conservation, and surface exposure.

**Figure 2:**
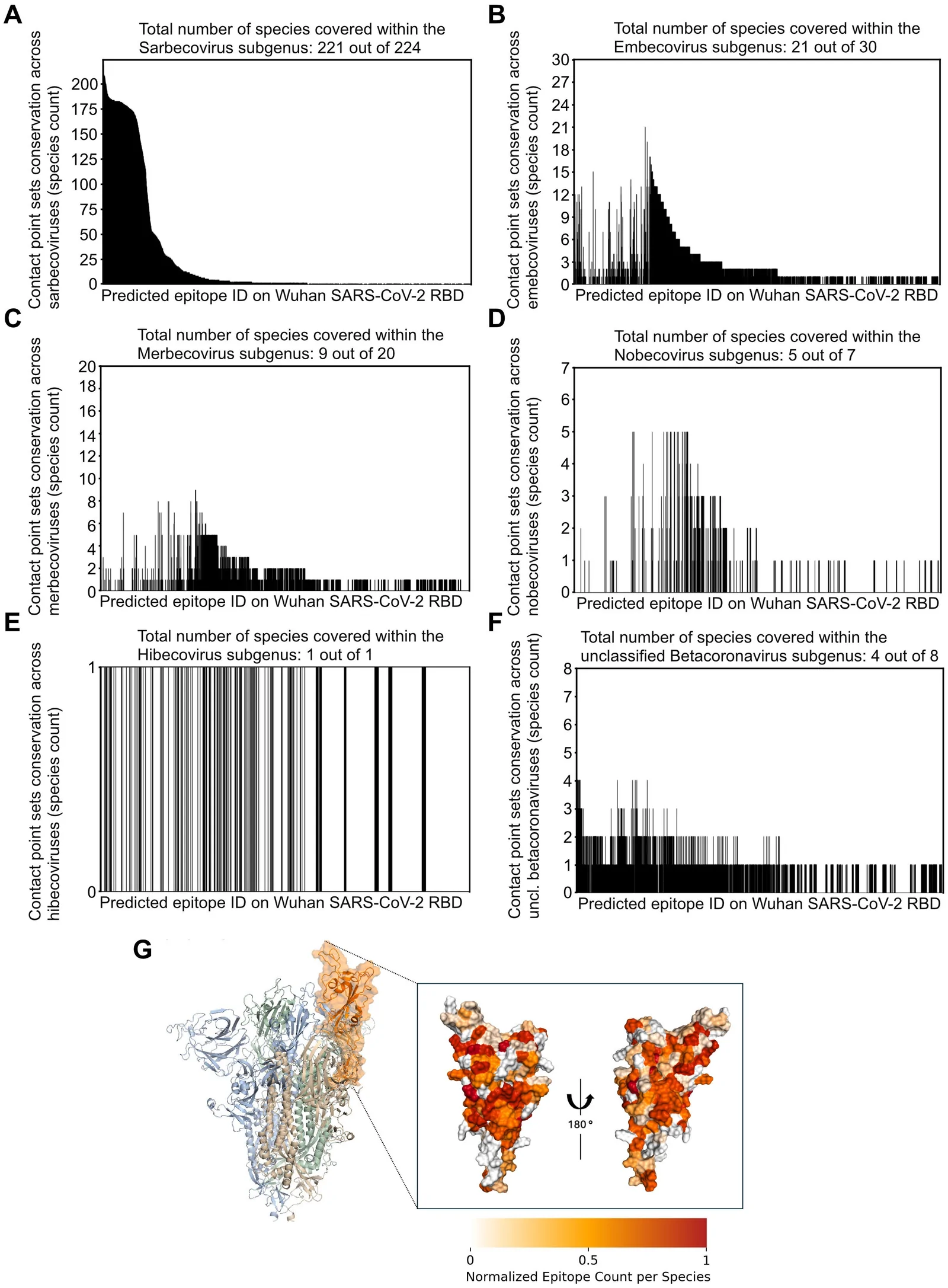
Conserved conformational epitope mapping across betacoronavirus subgenera. **(A-F)** Species distribution of positional permutation vector (PPV)–based conformational epitope predictions across major betacoronavirus subgenera. PPVs were defined within the SARS-CoV-2 receptor-binding domain (RBD) and mapped onto structurally aligned RBD regions of 290 betacoronavirus sequences (from 304 total Spike proteins). Each PPV identifier on the x-axis represents a unique conformational epitope candidate evaluated across aligned species. The y-axis indicates the number of species in which the predicted epitope probability exceeded 0.9. Subgenera shown are Sarbecovirus **(A)**, Embecovirus **(B)**, Merbecovirus **(C)**, Nobecovirus **(D)**, Hibecovirus **(E)**, and unclassified betacoronaviruses **(F)**. Heatmap of PPV distribution on the RBD of the SARS-CoV-2 **(G)**.

### 2.2 Epitope guided design of BA.2 spike immunogens

Building on the conserved epitope landscape identified across betacoronaviruses, we next designed engineered Spike immunogens enriched with epitopes conserved across species and starting from the SARS-CoV-2 BA.2 Omicron sequence. BA.2 was selected as the design backbone because it was the dominant circulating variant at the time of study and captures antigenic features of recent viral evolution, including mutations associated with immune escape. Using this sequence as a starting point enabled the design of immunogens that remain compatible with contemporary viral strains while incorporating conserved elements to promote broader cross-reactivity. Four epitope positions within the BA.2 RBD were selected where the native BA.2 motif differed from the most conserved motif observed across betacoronavirus species (**Figures 3A–D**; **Supplementary Figure 5**). For each position, the BA.2 sequence was mutated to match the most frequent epitope motif identified in the previous step. For example, at one site the native BA.2 motif APGQ was replaced with the conserved motif TPGQ by introducing the A373T substitution, generating an *in silico* variant predicted to promote cross-reactive antibody responses (**Figure 3A**). Importantly, these short motifs represent spatially proximal but non continuous residues within the RBD (e.g., APGQ corresponds to A373, P409, G410, and Q411), reflecting conformational epitope rather than isolated linear epitopes. To further maximize strain coverage, we explored additional single amino-acid substitutions within the conserved motifs and systemically recalculated epitope probabilities across all betacoronavirus species (**Figures 3E–H**). For each site, the mutation that maximized the number of species exceeding the epitope probability threshold (>0.9) was selected (**Figures 3I–L**). This iterative optimization yielded four engineered BA.2-based Spike constructs (denoted as Spike BA.2 NEC 1-4), each containing a distinct conformational epitope-focused mutation designed to increase the breadth of predicted antibody recognition across diverse betacoronaviruses. To ensure that these epitope-driven modifications did not compromise structural integrity, we evaluated the conformational stability of the engineered BA.2 Spike trimers relative to the wild-type BA.2 Spike using the Rosetta all-atom energy calculations across 20 independent conformations per construct (**Figure 3M**). All four Spike BA.2 NEC variants displayed energy scores that were comparable to or more favorable than wild-type BA.2 (more negative scores indicating increased predicted stability), with mean Rosetta energies of −9194.4 (Spike BA.2 NEC 2), −9193.0 (Spike BA.2 NEC 4), −9187.7 (Spike BA.2 NEC 1), and −9166.3 (Spike BA.2 NEC 3), compared to −9125.1 for the wild-type BA.2 Spike. These results indicate that the introduced mutations did not destabilize the trimeric Spike folding. Consistent with these findings, molecular dynamics simulations of the glycosylated RBDs for wild-type BA2 and each designed variant revealed similar root-mean-square deviation (RMSD) (**Supplementary Figure 1**) and root-mean-square fluctuation (RMSF) (**Supplementary Figure 2**) profiles between the engineered constructs and the wild-type BA.2 RBD, furthermore local RMSF in the region 360-370 of Spike, where mutations were introduced was significantly reduced. These observations suggest that the introduced mutations maintain native-like conformational behavior locally and across the full-length antigen.

**Figure 3:**
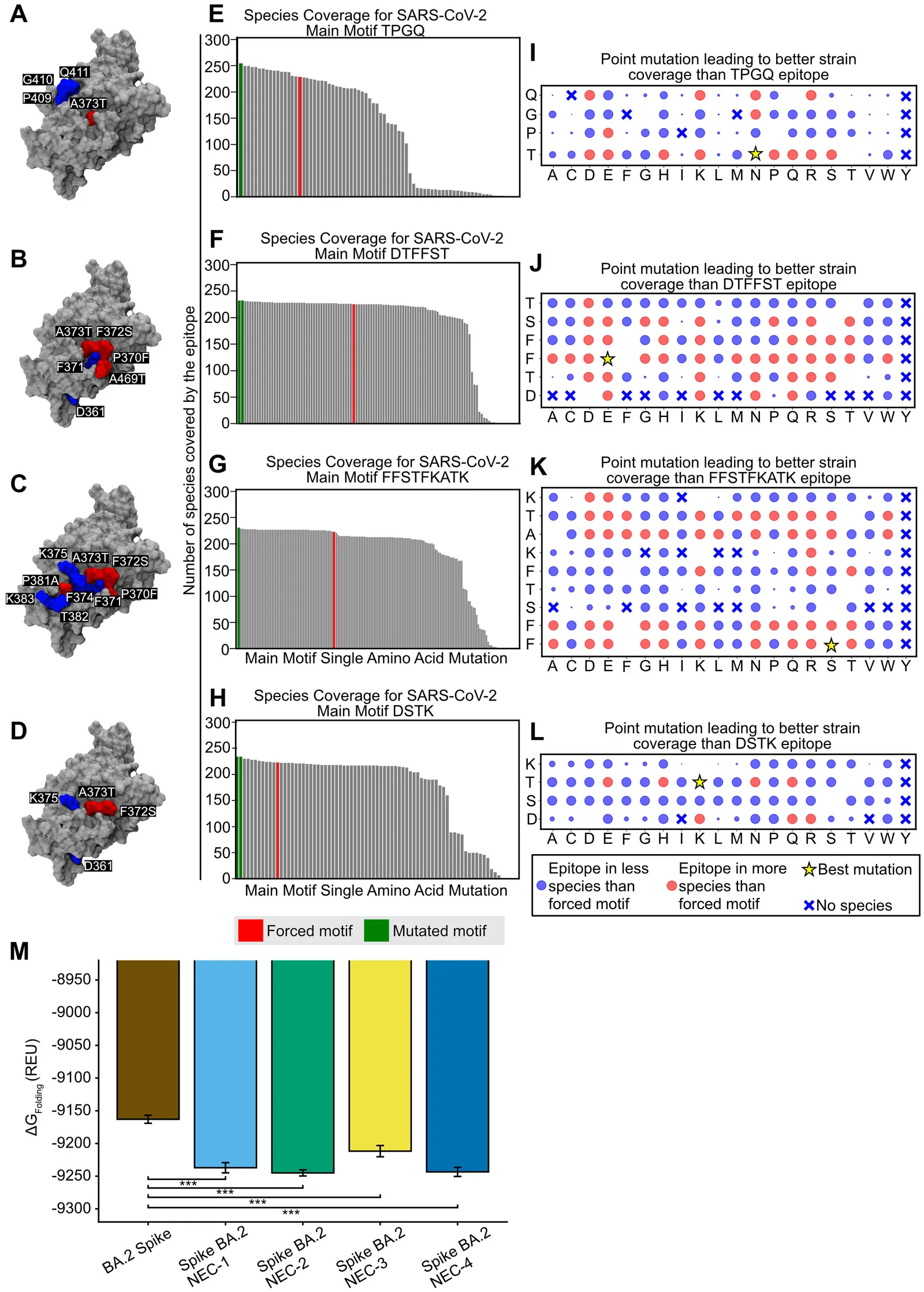
Epitope-guided engineering and structural evaluation of BA.2 Spike variants. **(A–D)** Structural mapping of selected epitope positions within the BA.2 RBD. Three-dimensional representations of the RBD show sites where the native BA.2 motif differs from the most frequent motif identified across betacoronaviruses. Highlighted residues indicate substitutions introduced to convert the BA.2 sequence to the conserved motif at each position. **(E–H)** Systematic evaluation of motif substitutions. For each PPV–defined epitope, candidate single–amino acid substitutions were introduced and re-evaluated using the conformational B cell epitope predictor across aligned strains. **(I–L)** Species coverage following motif optimization. For each engineered substitution, the number of strains with predicted epitope probability >0.9 is shown, and the mutation maximizing coverage was selected. **(M)** Rosetta all-atom energy analysis of wild-type and engineered BA.2 Spike trimers (Spike BA.2 NEC 1–4). Energy scores were calculated across independent conformations; more negative values indicate lower predicted energy.

### 2.3 Engineered spike vaccines induce broad IgG responses

To experimentally assess whether the engineered Spike proteins translate into broader and more potent humoral immunity *in vivo*, the four Spike BA.2 NEC constructs were encoded into mRNA vaccines and formulated in lipid nanoparticles. These experimental vaccines were administered to mice alongside comparator formulations encoding the ancestral full-length Wuhan Spike, the licensed bivalent Comirnaty Original + BA.4-5 (containing tozinameram and famtozinameram, respectively encoding for the ancestral and the BA.4/BA.5 full length Spike, both in prefusion stabilized form carrying the K986P/V987P ‘2P’ substitutions) vaccine as a clinical benchmark, and a GFP mRNA control. This experimental design enabled a direct comparison of epitope-engineered Spike immunogens with both an established strain-updated vaccine and a conventional full-length Spike antigen. To comprehensively evaluate vaccine-induced antibody responses, we quantified antigen-specific IgG responses by ELISA and assessed functional neutralizing activity against a panel of SARS-CoV-2 variants and related zoonotic sarbecoviruses using live-virus neutralization assays.

To compare antigen-specific IgG responses across vaccination groups and time points, ELISA OD450–OD630 values were normalized to the median signal of unvaccinated animals at day 42 for each antigen and expressed as log_2_ fold-change. The resulting values were visualized as a heatmap, enabling direct comparison of the relative magnitude and consistency of humoral responses across groups and time points. Overall, all antigen-encoding vaccine constructs induced robust IgG responses against SARS-CoV-2 prototype, BA.4/BA.5, and XBB.1.5 compared to unvaccinated and GFP controls (**Figure 4A**). Importantly, the Spike BA.2 NEC vaccine constructs consistently elicited strong antigen-specific IgG responses at both Day 42 and Day 63. For example, at Day 63 post vaccination, Spike BA.2 NEC 1 induced median log_2_ fold-changes of ~6.5, ~6.2, and ~6 against the prototype, BA.4/BA.5, and XBB.1.5 antigens, respectively, relative to unvaccinated baseline. Similarly, Spike BA.2 NEC 2-4 showed median log_2_ fold-changes ranging from ~5.8 to ~7.1, indicating robust and broad reactivity across variants. The magnitude of the IgG responses induced by the Spike BA.2 NEC constructs was comparable to, and in several antigen-group combinations exceeded, that observed for the full-length Spike LNP control (median log_2_ fold-changes of ~5.5-6.8 at Day 63 across antigens). Antibody levels were largely stable between Day 42 and Day 63 for all Spike BA.2 NEC constructs, with only minor changes in median log_2_ fold-change (typically < 0.3 log_2_ units), indicating sustained humoral responses over the interval assessed (Days 42-63). The Comirnaty (prototype + BA.4/BA.5) group was included as a clinical benchmark and showed high and stable IgG responses across all three antigens (median log_2_ fold-changes of ~6.7-6.9 at both time points), providing a reference point for the magnitude and short-term stability of the responses achieved by the Spike BA.2 NEC vaccine candidates over the interval assessed.

**Figure 4:**
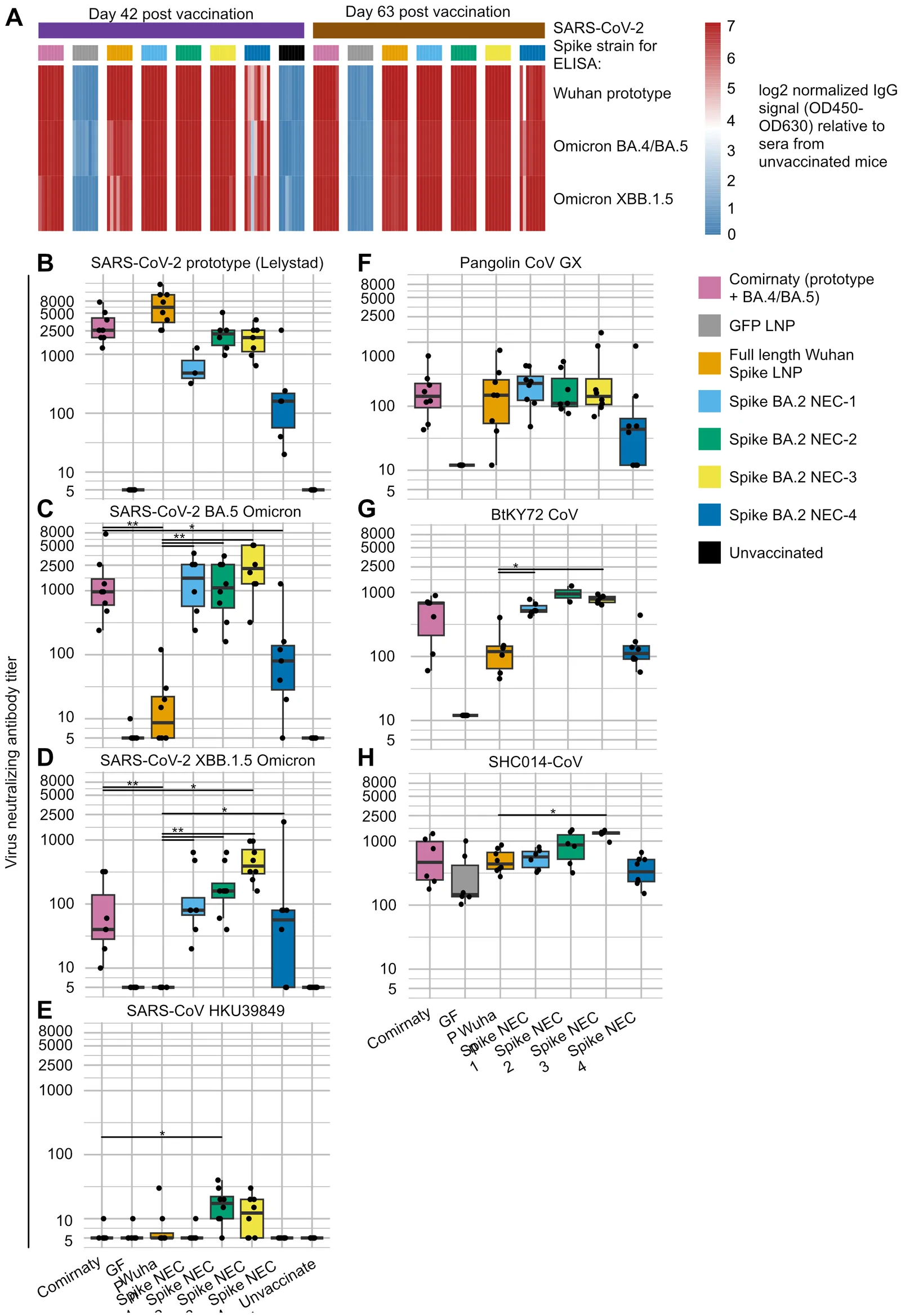
Vaccination with Spike NEC constructs induces cross-protective antibodies against multiple strains *in vivo*. **(A)** Antigen-specific serum IgG responses measured by ELISA. Heatmap showing normalized IgG binding against SARS-CoV-2 prototype, Omicron BA.4/BA.5, and Omicron XBB.1.5 receptor-binding domains. Optical density (OD450–OD630) values were normalized to the median response of unvaccinated animals at Day 42 for each antigen and expressed as log_2_ fold-change. Columns represent individual mice, annotated by vaccination group and sampling day (Day 42 and Day 63). **(B–E)** Live-virus neutralization titers after prime–boost immunization (Days 0 and 28; sera collected Day 42). Neutralizing antibody titers against SARS-CoV-2 prototype (Wuhan) **(B)**, Omicron BA.5 **(C)**, Omicron XBB.1.5 **(D)**, and SARS-CoV HKU-39849 **(E)** were determined by immunoperoxidase monolayer assay. Titers are shown as reciprocal log_2_ values of the highest serum dilution inhibiting infection in ≥50% of wells. **(F–H)** Live-virus neutralization titers after prime–double boost immunization (Days 0, 28, and 56; sera collected Day 70). Neutralization against Pangolin-CoV GX **(F)**, BtKY72 **(G)**, and SHC014 **(H)** is shown. For all neutralization panels, points represent individual mice; boxplots indicate median and interquartile range. Statistical comparisons were performed using pairwise Wilcoxon tests with Benjamini–Hochberg correction for multiple testing.

Paired statistical testing between Day 42 and Day 63 within each vaccination group revealed no substantial increase in IgG titers over time for most antigen–group combinations, suggesting that antibody levels had largely plateaued by Day 42 and were maintained through Day 63. This stability was particularly evident for the Comirnaty group, which showed nearly identical median normalized IgG levels at both time points across all tested antigens.

Across antigens and time points, Spike BA.2 NEC vaccine constructs elicited robust antigen-specific IgG responses that were comparable to or exceeded those induced by full-length Spike LNP and the Comirnaty reference vaccine. While overall response magnitudes were within a similar range across vaccine modalities, several Spike BA.2 NEC constructs demonstrated significantly higher IgG titers than full-length Spike LNP for the BA.4/BA.5 and XBB.1.5 antigens, highlighting the potential advantage of epitope-mutagenesis vaccine designs for variant-proof humoral immunity. At day 42, Spike BA.2 NEC 2 induced significantly higher BA.4/BA.5-specific IgG responses compared with full-length Spike LNP (Dunn’s test, adjusted p = 0.0098), and Spike BA.2 NEC 4 showed higher responses than both Spike BA.2 Spike BA.2 NEC and Spike BA.2 NEC 2 for the SARS-CoV-2 prototype antigen (adjusted p = 0.021 and 0.046, respectively). Notably, at day 63, multiple Spike BA.2 NEC constructs outperformed full-length Spike LNP for the XBB.1.5 antigen: Spike BA.2 NEC 1 (padj = 0.0038), Spike BA.2 NEC 3 (padj = 0.0167), and Spike BA.2 NEC 4 (padj = 0.0020) all elicited significantly higher IgG responses than the full-length Spike LNP control. In contrast, Comirnaty and full-length Spike LNP did not consistently outperform Spike BA.2 NEC constructs across variant antigens, indicating that Spike BA.2 NEC designs can match or surpass established vaccine formats in inducing variant-directed humoral responses.

### 2.4 Broad neutralization of SARS-CoV-2 variants and sarbecoviruses

Neutralizing antibody titers were assessed against SARS-CoV-2 prototype (Wuhan), BA.5, XBB.1.5, SARS-CoV (HKU), BtKY72 bat coronavirus, and pangolin coronavirus (**Figures 4B–H**). Titers against SARS-CoV-2 prototype, BA.5, XBB.1.5 and HKU were measured after a prime-boost regimen (Day 42), whereas BtKY72 and pangolin CoV neutralization was assessed after a prime-double boost regimen (Day 70). Neutralization titers are reported in the main text for clarity, while full descriptive statistics are provided in **Supplementary Tables 1**, **2**.

Against the SARS-CoV-2 prototype (**Figure 4B**), all vaccine formulations induced high neutralizing activity. The ancestral full-length Spike elicited the highest titers, while Comirnaty and NEC-2/NEC-3 achieved comparable responses, indicating preserved neutralization capacity of these constructs against the ancestral strain. In contrast, NEC-1 and particularly NEC-4 showed reduced activity. However, these differences did not reach statistical significance after multiple testing correction.

A marked divergence emerged against BA.5 (**Figure 4C**). While Comirnaty maintained robust neutralization, the ancestral Spike vaccine showed near-background activity, consistent with antigenic mismatch. In contrast, NEC-1, NEC-2, and NEC-3 retained strong neutralizing responses, significantly exceeding the ancestral Spike vaccine (adjusted p ≤ 0.008), and reaching levels comparable to Comirnaty. NEC-4 showed substantially weaker activity, remaining significantly below Comirnaty (p.adj = 0.023). This pattern was further accentuated against XBB.1.5 (**Figure 4D**), where the ancestral Spike failed to induce detectable neutralization. All NEC constructs retained activity, with NEC-3 showing the strongest response and significantly exceeding both the ancestral Spike (p.adj ≤ 0.028) and Comirnaty (p.adj = 0.028). Comirnaty itself exhibited only limited neutralization, highlighting the increased antigenic distance of this variant.

Cross-neutralization of SARS-CoV (HKU) remained limited overall (**Figure 4E**) but was detectable for NEC-2 and NEC-3, which showed significantly higher titers than Comirnaty and NEC-4 (p.adj ≤ 0.048), indicating that cross-reactive responses are construct-dependent rather than a general feature of all designs. In the prime–double boost setting, neutralization of pangolin CoV was comparable across groups, with no significant differences observed. In contrast, against BtKY72, NEC-1 and NEC-3 elicited significantly higher titers than the ancestral Spike (p.adj = 0.012) and NEC-4, indicating improved cross-neutralization of divergent sarbecoviruses. A similar trend was observed for SHC014, where NEC-3 induced the highest responses and significantly outperformed both ancestral Spike and lower-performing constructs.

Overall, NEC-2 and NEC-3 consistently demonstrated the most favorable neutralization profiles, maintaining activity across antigenically drifted SARS-CoV-2 variants and extending neutralization to zoonotic sarbecoviruses, reaching neutralization levels comparable to the variant-adapted Comirnaty benchmark for shared SARS-CoV-2 antigens. Comirnaty was included as a clinical reference point, differing from the in-house constructs in both dose and LNP formulation, comparisons to Comirnaty are to be interpreted as contextualization against an approved clinical product rather than an absolute experimental comparator. The experimental comparator is the full-length wild-type Wuhan Spike control, against which all design-related conclusions are drawn.

### 2.5 Rational epitope engineering enhances cross-reactive neutralization

To mechanistically relate epitope engineering to the observed breadth of neutralization, we performed *post hoc* epitope probability analyses across the viral strains included in the neutralization assays. Importantly, during the initial design phase, epitope selection was based on a single representative sequence per virus species; for SARS-CoV-2, this corresponded to the prototype strain. Variant-specific sequences such as BA.5 and XBB.1.5 were therefore not explicitly incorporated during design. To assess how the engineered motifs generalize beyond this design space, we compared predicted B-cell epitope probabilities of the original strain-specific motifs with those of the engineered motifs across divergent variants.

Across all constructs and viruses tested, the introduced mutations consistently increased predicted epitope likelihood, frequently converting motifs with low or negligible immunogenic potential into high-probability epitopes (**Figures 5A–D**). For example, in Spike BA.2 NEC-1, the BA.5 and XBB.1.5 motif APGQ showed low predicted probabilities (0.21 and 0.093), which increased to 0.935 and 0.939 following substitution with the engineered NPGQ motif. Similar improvements were observed for Spike BA.2 NEC-2, where the BA.5 motif DAPFFA (0.051) increased to 0.986 after mutation, and for Spike BA.2 NEC-3, where motifs with near-zero predicted probability (0.002) were converted into highly probable epitopes (>0.97). Importantly, for strains in which the original motif was already highly immunogenic (e.g., Wuhan), the engineered motifs preserved high predicted probabilities (≥0.99), indicating that epitope optimization did not compromise recognition of conserved antigenic features. These results demonstrate that the introduced mutations selectively enhance epitope visibility across antigenically divergent variants while maintaining compatibility with conserved epitopes.

**Figure 5:**
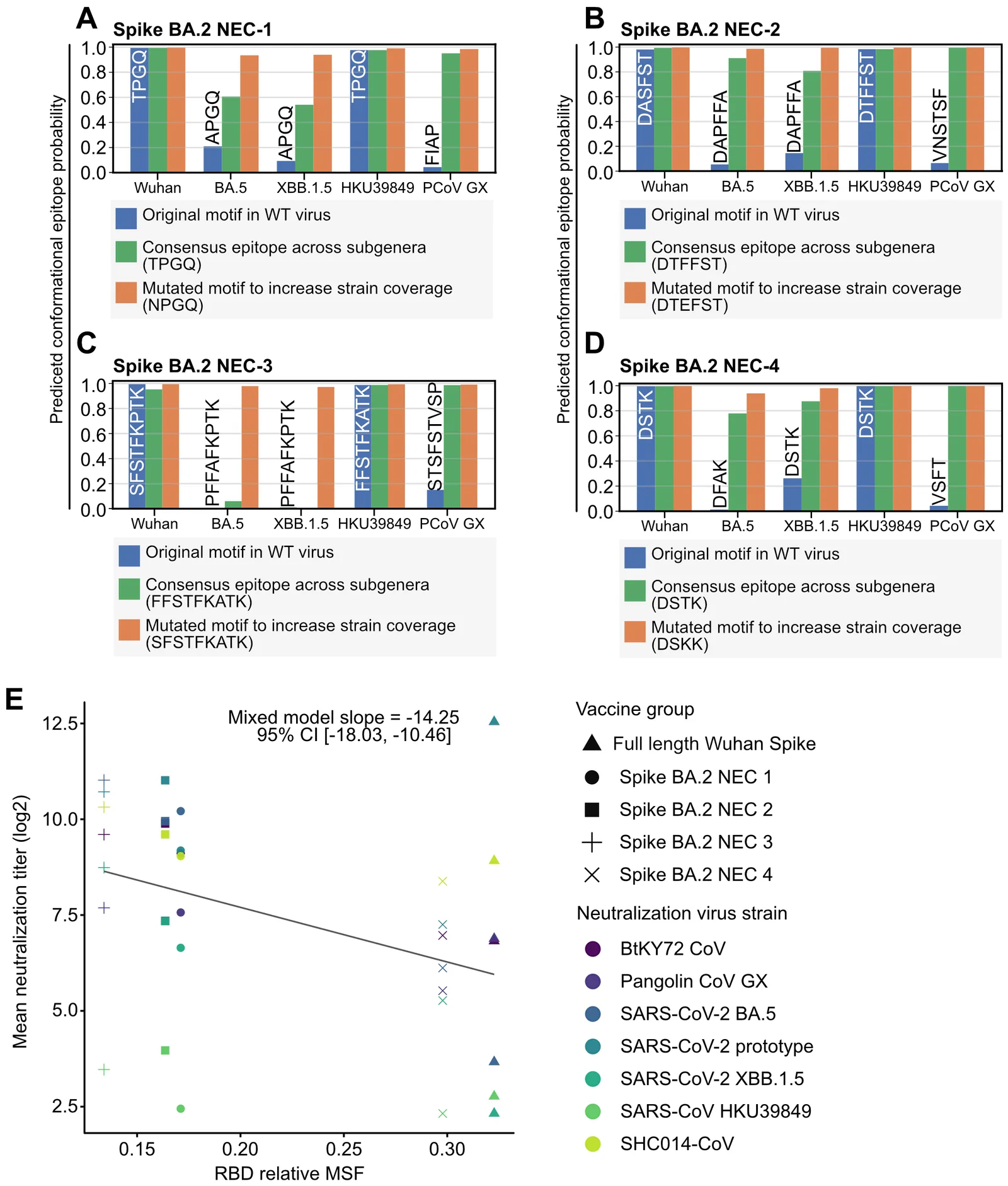
Epitope probability remodeling and structural correlates of neutralization breadth. **(A–D)** Predicted conformational B cell epitope probabilities for original, conserved, and engineered motifs at selected RBD positions in Spike BA.2 NEC 1–4. For each construct, bar charts show epitope probabilities across viral strains used in neutralization assays, comparing (i) the native strain-specific motif, (ii) the most frequent motif across betacoronaviruses, and (iii) the engineered motif introduced into the BA.2 Spike backbone. Probabilities were calculated using the PPV-based B cell epitope predictor. **(E)** Association between RBD conformational flexibility and neutralization titers. Mean log_2_ neutralization titers for each vaccine construct and virus strain are plotted against the RBD flexibility from ENM analysis within the full spike. The line represents the fixed-effect estimate from a linear mixed-effects model fitted using restricted maximum likelihood, with log_2_ neutralization titers as the response variable, RBD relative MSF as a fixed effect, and random intercepts for vaccine construct and virus strain.

To further link epitope optimization to functional antibody breadth, we examined the relationship between neutralization titers and RBD conformational dynamics within the context of the full trimeric Spike, quantified via elastic network model (ENM) analysis as a domain-averaged mean squared fluctuation of RBD residues (hereafter, RBD MSF). Neutralization breadth inversely correlated with RBD flexibility, with linear mixed-effects modeling indicating that lower relative RBD MSF was associated with higher neutralization titers across strains and vaccine constructs (estimate = −14.25, t = −7.38; **Figure 5E**). Consistent with this relationship, constructs exhibiting stronger cross-neutralization, such as NEC-2 and NEC-3, also displayed reduced RBD fluctuations, suggesting that stabilization of the RBD contributes to improved antibody recognition across diverse viral targets.

Consistent with these observations, broader constructs also exhibited enhanced RBM–FP allosteric coupling, which was lowest for Wuhan Spike and NEC-4 (0.068 and 0.070, respectively) and higher in constructs with improved cross-reactivity (NEC-2: 0.079; NEC-1 and NEC-3: 0.076; **Supplementary Figure 3**), indicating more effective long-range communication within the Spike protein. While global RBD solvent accessibility profiles were comparable across constructs, NEC-1 and NEC-4 showed increased exposure at residue 373, and electrostatic surface analysis revealed a relative shift toward electropositive character in NEC-4 (**Supplementary Figure 3**). These structural features provide a plausible explanation for the reduced cross-reactivity observed for NEC-4, linking localized epitope presentation and surface charge distribution to functional antibody breadth.

Because all four Spike BA.2 NEC constructs share an identical backbone and were produced with identical mRNA chemistry, LNP formulation, dose and immunisation regimen, differing solely in the single-residue substitution at a distinct epitope position, this construct-dependence is directly attributable to the specific epitope engineering. The predicted conformational B-cell epitope probability against SARS-CoV (HKU) exceeds 0.9 for the engineered motifs across all four constructs (**Figures 5A–D**). The selective cross-neutralization of HKU by NEC-2 and NEC-3 therefore reflects not differential sequence-level epitope enrichment but their more favourable conformational profiles, reduced RBD MSF and stronger RBM–FP allosteric coupling.

## 3 Discussion

The objective of this study was to develop a rational, epitope-focused Spike engineering strategy that enhances the presentation of conserved, cross-reactive conformational epitopes within a single Spike backbone, with the aim of broadening antibody responses across antigenically diverse sarbecoviruses without relying on strain-specific sequence updates. Using a structure-informed epitope prediction framework applied to aligned betacoronavirus RBDs, we identified conserved conformational hotspots and introduced minimal motif-level substitutions into a BA.2 Spike background while maintaining predicted structural stability. The key new finding is that these engineered BA.2 Spike mRNA-LNP vaccines elicited robust IgG responses and broadened live-virus neutralization across SARS-CoV-2 variants and divergent zoonotic sarbecoviruses compared with an ancestral full-length Spike vaccine. Although experimental validation in this study was restricted to the sarbecovirus subgenus, the engineered immunogens were designed using a betacoronavirus-wide epitope-conservation framework from the outset: the conformational epitope motifs introduced into the NEC constructs were selected precisely because they are predicted conserved across all major betacoronavirus subgenera: Embecovirus, Merbecovirus, Nobecovirus and Hibecovirus, as well as Sarbecovirus (**Figure 2**). The designs therefore already encode cross-subgenus conserved determinants, what remains unaddressed is whether this translates into cross-neutralizing antibody responses against non-sarbecovirus betacoronaviruses, including MERS-CoV and endemic human betacoronaviruses. Notably, selected constructs (Spike BA.2 NEC2/3) reached neutralization breadth comparable to an Omicron-updated clinical benchmark for neutralizing breadth against BA.5 and XBB.1.5, supporting structure-guided epitope optimization as a generalizable route to more variant-resilient coronavirus vaccines. This brings possibility of using novel mechanism to engineering vaccines by changing the “readout” of the antigen by the immune system, rather than relying only on reactive strategies such as updating the vaccine to specific emerging strains or using multi-antigen formulations (30, 31).

Recent efforts to achieve pan-sarbecovirus immunity have explored multivalent and mosaic nanoparticle vaccines that co-display RBDs from multiple strains to bias B cell responses toward conserved epitopes (30, 31). In these platforms, breadth is achieved by presenting heterologous antigens on the same particle to favor avidity-driven recognition of shared determinants while limiting efficient cross-linking of strain-specific epitopes (32, 33). While this strategy has demonstrated encouraging cross-protection in preclinical models, it relies on the inclusion of predefined viral sequences and entails increased manufacturing complexity associated with multicomponent antigen assembly (34). In contrast, our approach does not depend on combinatorial antigen display but instead enhances the presentation of conserved epitopes within a single Spike backbone through targeted epitope engineering. By enhancing conserved RBD determinants while preserving structural stability, we achieve broad neutralization using a minimal set of defined mutations encoded in a single mRNA construct. This structurally guided antigen redesign represents a complementary and potentially more scalable strategy for inducing pan-sarbecovirus immunity without requiring multivalent formulation.

An alternative strategy to broaden sarbecovirus immunity has been the use of modular chimeric Spike constructs in which RBD, N-terminal domain (NTD), and the subunit 2 (S2) from different viruses combined within multiplexed mRNA formulations (35). These designs demonstrated encouraging cross-protection not only for SARS-CoV-2 but also for influenza, underscoring the feasibility of expanding antigenic coverage by domain substitution (36, 37). However, breadth in this context is achieved through combinatorial inclusion of heterologous domains rather than through enhancement of conserved-epitope presentation, and multiplexed administration introduces potential antigen competition, reduced strain-matched neutralization potency and potentially an antigenic sin (35, 38). In addition, co-expression of multiple Spike variants raises the possibility of heterotrimer formation and increased manufacturing complexity, which may complicate translational scalability (39). In contrast, our approach maintains a single Spike backbone and achieves breadth through minimal, structure-guided epitope remodeling that enhances conserved RBD determinants while preserving conformational stability. By directly reshaping the antigenic surface rather than combining divergent domains, this strategy provides a straightforward manufacturing and scalability strategies.

Bivalent mRNA vaccines represent an important step toward broadening immunity against antigenically drifted SARS-CoV-2 variants. Notably, booster immunization with bivalent mRNA-1273.214 or mRNA-1273.222 increased neutralization breadth and reduced viral burden and lung pathology following BA.5 challenge in mice (21). However, mechanistic analyses revealed that a substantial proportion of the anti-Omicron RBD response after boosting remained cross-reactive with the ancestral Wuhan spike, reflecting shaping by prior immune memory rather than exclusively *de novo* targeting of variant-specific epitopes (21). As seen in influenza, the antigenic sin can imprint the response toward epitopes encountered during initial exposure, often at the expense of generating a robust *de novo* response for the new variant (40). Thus, while bivalent updates increase breadth within the existing antigenic landscape, they may remain constrained by prior immunological encounters with pathogen or vaccines. In contrast, our strategy seeks to proactively enhance conserved-epitope presentation at the antigen design level, independent of strain inclusion or antigenic sin. Because the targeted determinants are shared across sarbecoviruses and immunologically compatible with ancestral Wuhan-elicited memory, we hypothesise that this approach may be less susceptible to imprinting-driven suppression than strategies that introduce novel variant-specific epitopes, a design rationale that remains to be tested experimentally in a pre-immunized setting.

A conceptually related strategy was recently described by Vishwanath et al., who designed multi-epitope Spike immunogens by grafting sets of naturally occurring variant-of-concern mutations onto a Wuhan-Hu-1 backbone, with epitope sites identified from linear immunodominant regions defined by the IEDB database and the peptides reported by Lu et al. (41, 42). While sharing the objective of broadening humoral immunity within a single immunogen, our approach differs in three respects: (1) we predict conformational B cell epitopes rather than linear database-annotated sites; (2) we introduce minimal single-residue substitutions rather than whole sets of observed variant mutations across multiple spike domains that might compromise the protein structure and stability; (3) and our substitutions are residues selected for predicted conservation across the betacoronavirus genus, rather than mutations already observed in circulating human SARS-CoV-2 variants. In a nutshell, while Vishwanath et al. optimise within known antigenic space, our conformational epitope-centric approach is designed to be predictive, targeting conserved determinants that may pre-empt future antigenic drift (42).

Notably, these approaches are not mutually exclusive, our antigen design strategy could in principle be integrated into multivalent or mosaic platforms, to further enhance strain resilience, or even to make combination vaccines against multiple pathogens.

The preserved structural stability observed in our engineered variants has critical implications for immunogen design. The maintenance of native-like flexibility profiles, as evidenced by similar RMSD/RMSF patterns, ensures that critical conformational epitopes remain accessible while avoiding the rigidification that can compromise antigenicity (43). This balance between stability and conformational dynamics is particularly crucial for Spike-based immunogens, where preserved receptor binding domain mobility facilitates cross-reactive antibody recognition across variant strains (44). Our findings suggest that epitope-guided modifications can enhance immunogen stability without sacrificing the conformational plasticity to avoid tight locking the antigen into a rigid state that restricts the access to relevant conserved epitope required for broad neutralizing responses.

Importantly, epitope prediction and stability assessment are independent steps in our pipeline. The BCE predictor scores antibody-contact probability and does not model folding stability. The substitutions introduced are selected for predicted epitope conservation, not for stabilisation. Structural stability is evaluated separately (Rosetta energy and molecular dynamics), and candidate substitutions predicted to destabilise folding were discarded during design. The observed structural integrity of the final constructs is therefore an independently assessed property rather than a feature promoted by the epitope-prediction framework.

Within these limitations, the data suggest that the engineered mutations in the NEC spikes strengthen the mechanical pathway connecting the RBM to the fusion peptide, and that enhanced allosteric communication is associated with broader cross-neutralization. Tighter RBM–FP coupling may reflect a more coordinated conformational landscape in which the RBD samples states that expose conserved epitopes more effectively upon immune recognition. This would be consistent with the view of the spike as a dynamic allosteric machine whose conformational equilibrium directly influences immunogenicity (45, 46).

The relationship between epitope engineering and cross-neutralization breadth is most clearly illustrated by the internal-control structure of the construct panel. The four Spike BA.2 NEC immunogens form an isogenic series differing only in the single-residue epitope substitution; any difference in cross-neutralization between constructs is therefore causally attributable to the epitope engineering alone. That all four constructs carry a high-probability HKU-relevant epitope (>0.9; **Figures 5A–D**) yet only NEC-2 and NEC-3 cross-neutralize HKU indicates that epitope probability and RBD conformational dynamics are complementary, jointly necessary determinants of cross-reactive humoral immunity.

This immunogen engineering strategy can be used beyond betacoronavirus subgenera to other pathogens where the vaccine faces similar constraints such as immunodominance, antigenic sin and emerging new variants seasonally. Seasonal influenza vaccine strain recommendations are made months before peak circulation to allow manufacturing and distribution (19, 47). The antigenic drift reduces the vaccine-virus match to thus leading to a vaccine efficacy of roughly 40% (19). To improve current strain-selection frameworks, epitope-guided antigen redesign could be integrated into a modular pipeline in which genomic surveillance and evolutionary forecasting define plausible antigenic trajectories, structural constraint analyses prioritize functionally conserved sites, and rational engineering enhances the presentation of cross-reactive regions of the antigen while preserving structural stability and native-like dynamics. In influenza, this approach could reduce the penalty imposed by drift between strain selection and circulation by biasing responses toward highly variable epitopes.

Despite positive results on broad Spike immunogenicity, our study is associated with several limitations. First, the absence of a direct control group against the BA.2 variant constrains our ability to distinguish variant-specific effects from broader patterns of neutralization, and therefore limits the strength of inferences regarding the relative performance of the framework in that antigenic background. Second, the structure–activity relationships described here should be interpreted cautiously, as they are derived primarily from *post hoc* associations rather than from a prospective design or experimental perturbation strategy capable of establishing causality. Accordingly, these observations are best viewed as hypothesis-generating rather than definitive mechanistic conclusions. Third, the generalizability of the approach may be restricted by the fact that Spike represents a comparatively tractable test case. In particular, the receptor-binding domain is intrinsically enriched for antibody-targeted neutralizing epitopes, creating a structural and functional landscape in which neutralization is more likely to be captured. This favorable property is not explicitly modeled by our tool, and performance may therefore not extrapolate directly to antigens or protein regions in which the relationship between local residue features and neutralizing potential is less pronounced. Together, these considerations indicate that further validation across variants, antigens, and experimentally controlled settings will be necessary to define the scope and robustness of the approach.

A further limitation is that immunogenicity was assessed only through serum IgG binding and serum neutralization; mucosal antibodies, in particular secretory IgA, were not evaluated. This is in line with the intramuscular route employed, which predominantly induces systemic humoral immunity with limited respiratory-mucosal IgA (48, 49), whereas the serum neutralizing titers measured here are an established correlate of protection against symptomatic and severe disease (50, 51). Because mucosal IgA contributes to blocking viral entry and reducing transmission, such a broadly cross-reactive immunogen as we have designed might be well suited for mucosal administration if coupled with an appropriate delivery system. Engineered immunogens have been previously shown to generate cross-reactive mucosal immunity across sarbecoviruses (52).

In addition, the immunological readouts in this study extend only to approximately three weeks after the final immunization (Day 63 for binding IgG and Day 70 for neutralisation). The durability of the responses was therefore not assessed, and our data do not address whether the approach mitigates the waning of immunity observed with current vaccines. Establishing durability will require longitudinal follow-up over several months, together with characterization of the memory B-cell and long-lived bone-marrow plasma-cell compartments that determine the persistence of humoral immunity.

For logistical reasons, the most antigenically divergent sarbecoviruses (BtKY72 and SHC014-CoV) were evaluated after a third immunization (Day 70), whereas the remaining neutralization panel was assessed after two doses (Day 42); absolute titers across the two panels should not be compared directly. Critically, however, the third immunization was administered to all vaccine groups simultaneously, so any effect of additional dosing on response breadth would be expected equally across groups. The selective cross-neutralization of BtKY72 and SHC014-CoV by Spike BA.2 NEC-2 and NEC-3, but not by the Wuhan Spike control or the remaining NEC constructs, therefore reflects the epitope engineering rather than the immunization schedule. This conclusion is independently supported by the cross-neutralization of SARS-CoV (HKU) observed in the two-dose Day 42 cohort, which is free of any third-dose confound.

A limitation of the Comirnaty comparison is that this product was administered at a ten-fold lower dose and in a different proprietary LNP formulation, and was included as a clinical reference point rather than a matched experimental control. The design conclusions rest on the internally matched in-house comparisons, Spike BA.2 NEC-1 to NEC-4 and the full-length Wuhan Spike control, which shared identical mRNA chemistry, LNP platform, dose and regimen and differed only in the encoded antigen. The extension of neutralizing activity to divergent zoonotic sarbecoviruses reflects antigenic specificity rather than dose, since increasing the dose of a strain-restricted antigen cannot generate cross-neutralizing responses against strains whose conserved epitopes it does not encode. We further emphasise that Comirnaty was included to demonstrate that the in-house constructs elicit humoral responses within the range of a clinically available vaccine in the same model and assay system, a contextualisation exercise rather than a competitive comparison.

Finally, this study did not include a viral challenge experiment, limiting our ability to directly demonstrate protective efficacy *in vivo*. All experiments were conducted in immunologically naïve mice; the impact of pre-existing immunity and antigenic imprinting on the response to the engineered immunogens was therefore not assessed. Such studies remain challenging for SARS-CoV-2 and related sarbecoviruses because they require suitable animal models, strain-matched challenge viruses and a high-containment infrastructure that cannot be readily accessed to.

This work demonstrates the feasibility of an AI-guided conformational epitope engineering framework as a generalizable antigen design strategy, using the sarbecovirus Spike as a well-characterised and immunologically tractable model system. The central contribution is methodological, that a structure-informed, data-driven epitope prediction framework can translate conserved conformational B-cell epitope maps into minimally modified artificial antigens that broaden neutralizing antibody specificity across antigenically diverse viral strains. Because the framework operates on structural and sequence properties that are not unique to coronaviruses, the design principles demonstrated here have broad applicability beyond the sarbecovirus system explored here.

In summary, we demonstrate that minimal, structure-guided epitope remodeling within a single Spike backbone can broaden neutralization across antigenically diverse sarbecoviruses without compromising structural stability. By combining comparative epitope conservation analysis with rational mutagenesis and dynamic modeling, this work provides evidence that targeted motif-level edits can enhance the presentation of conserved epitopes and improve cross-neutralization breadth. These findings establish a framework for proactively engineering vaccine antigens toward conserved viral vulnerabilities rather than relying solely on reactive strain updates.

## 4 Materials and methods

### 4.1 B cell epitope prediction and construct generation

We used our recently published B-cell epitope predictor to identify conformational B-cell epitopes across the strains of each genus in silico (29). The predictor takes into account the conformational characteristics of the target protein and models candidate epitopes by encoding them as binary positional permutation vectors (PPVs). PPVs reveal the precise location of each candidate epitope on the protein, as well as the amino acids it is constituted from. The full structural span of predicted conformational epitopes with a probability greater than 0.9, encompassing both direct antibody-contact positions and the non-contact positions that form their conformational architecture, ranges from 4 to 215 amino acid residues, reflecting the discontinuous, three-dimensional nature of conformational epitopes. The direct antibody-contact positions within each PPV range from 4 to 16 residues, consistent with the prior that modelled the length and composition of experimentally identified antibody-antigen contact points in PDB. The distribution of epitope span and contact-point counts across all retained PPVs is provided in **Supplementary Figure 4**. Modelling each candidate epitope as a unique PPV enables focused analysis of individual epitopes by separating different epitopes from each other. Furthermore, the use of PPVs allows for the discovery of epitopes within specific regions of the target protein (e.g., RBS), the assessment of epitope coverage across different strains by using the same PPV for each, and the mutation of amino acids at specific positions on those epitopes.

To enable mapping of PPVs across diverse betacoronavirus strains, the 304 collected Spike protein sequences were aligned using a multiple sequence alignment with BLOSUM62 as the substitution matrix, of which 290 sequences could be aligned at the RBD level and were retained for downstream analysis. The resulting alignment established a shared residue-coordinate system, allowing each PPV, defined on the SARS-CoV-2 reference RBD, to be projected onto the homologous positions of every other aligned RBD. The same set of 450,000 candidate PPVs was then mapped onto each of the 290 aligned RBDs, yielding approximately 130 million PPV–strain combinations evaluated in total.

To discover shared epitopes across different SARS-CoV-2 strains, we generated a set of 450,000 unique candidate PPVs and mapped them onto the RBD region of each strain. Using pre-generated PPVs, shared across strains, we were able to identify possible shared epitopes within the RBDs and across strains. The candidate PPVs were generated through a prior that models the distribution of true epitopes and is built from Ab-Ag interaction data from the PDB database (29, 53). The B-cell predictor associates each PPV-strain combination with a probability of whether the PPV on that specific strain represents a true B-cell epitope. After running the B-cell predictor, we retained only PPVs with probabilities above 0.9 on at least one strain. These PPV-strain combinations reveal the true epitopes of each strain. A probability threshold of 0.9 was selected based on internal benchmarking analyses (29), which demonstrated that this threshold achieves optimal prediction accuracy, maximising specificity in conformational B-cell epitope identification while minimising false-positive epitope candidates.

We ranked the retained PPVs by the number of strains with which they are associated. Since each PPV encodes the positions of amino acids on the given strain, we used those to create sequence logos for each top-ranked PPV. These sequence logos revealed the underlying epitope sequence of each PPV across its associated strains, as well as a dominant epitope motif corresponding with that PPV. Moreover, each PPV and its corresponding sequence logo indicated the level of broad coverage across the strains, as reflected by their ranking.

To investigate the potential increase in broad coverage across the strains for each PPV, we proceeded in two steps. In the first step, we applied each dominant motif to strains that did not already have it, based on the corresponding PPV, which encodes the motif’s location. This procedure was performed for each top-ranked PPV; we then re-predicted with the B-cell predictor and counted the total number of strains for which each PPV-motif combination yielded a probability above 0.9. This created a baseline of coverage for each PPV-motif pair.

In the second step, we systematically mutated each amino acid of the motif to every other possible amino acid. Mutations were introduced one amino acid at a time, and each mutated motif was applied across all relevant strains based on the corresponding PPV. After each mutation, the B-cell predictor was used to recalculate probabilities, and the total number of strains for which the PPV-mutated motif exceeded a probability of 0.9 was counted. This process was repeated for all top-ranked PPV-motif pairs, generating a distribution of mutated motifs and the corresponding strains in which they were predicted as epitopes.

### 4.2 Structural modelling of antigens

Starting from the crystal structure of the closed state of the SARS-CoV-2 BA.2 spike head domain [PDB ID: 8D55 (54)], and SARS-CoV-2 Wuhan strain (PDB ID: 6VXX (44) with mutations added to match the used sequences in the experiments) the missing loops were modeled using the Rosetta next-generation kinematic closure (NGK) protocol (55) (Rosetta version 3.14). Loop sampling was guided by the ref2015 all-atom energy function (56) and carried out over five outer cycles, combining neighbor-dependent Ramachandran distributions, ω-angle sampling, and gradual ramping of steric repulsion (57). The clash threshold was set to 0.36 and the legacy kinematic closure implementation was disabled. Side chains were repacked and minimized with further diversity achieved through enhanced rotamer sampling. Bicubic interpolation of Ramachandran potentials was turned off (58). A total of 1,000 independent models were generated, and the structure with the lowest total score was further refined using Cartesian FastRelax with the *ref2015_cart* function (59, 60). Refinement consisted of four relaxation cycles, each allowing up to 200 minimization steps. To assess the effect of mutations on spike stability, four artificial variants were generated from the relaxed SARS-CoV-2 BA.2 spike structure. Each variant, along with the SARS-CoV-2 BA.2 spike control, was subjected to 20 independent FastRelax simulations with coordinate constraints on heavy atoms, each initiated from the same relaxed starting structure. Because coordinate-constrained FastRelax performs stochastic side-chain repacking and locally restrained backbone minimization around a shared starting conformation, this ensemble is designed to reduce stochastic scoring noise around a common local minimum rather than to sample distinct conformational substates; the same protocol, number of runs, and constraints were applied uniformly across all five constructs. From each set of 20 models, the average total score was computed. During the relaxation, interatomic interactions were included up to a 9 Å cutoff for the attractive van der Waals and solvation terms.

### 4.3 Molecular dynamics simulations

The crystal structure of the SARS-CoV-2 BA.2 RBD [PDB ID: 7ZF8 (61)] was used as the starting template. Point mutations were introduced using the Rosetta package, and each variant structure was relaxed using the Rosetta FastRelax protocol with coordinate constraints on heavy atoms (59, 60). Protonation states were assigned at pH 7.4 using PROPKA (62). A single N-glycan attached to ASN-343 was modelled as core-fucosylated biantennary structures (FA2) with terminal N-Acetylglucosamine residues, consistent with the predominant glycoform following mass spectroscopy data (63). Molecular dynamics simulations were performed with GROMACS 2022.5 (64) using the Amber ff14SB force field (65), GLYCAM06 parameters set for glycans (66), and TIP3P water model (67). Each system was solvated in a cubic box with an edge distance of 10 Å and periodic boundary conditions. Na^+^ and Cl^-^ ions were added to achieve electroneutrality and a buffer saline solution of 0.15 M. Particle Mesh Ewald was used for long-range electrostatics with a 10 Å Lennard-Jones cutoff (68). After energy minimization using the steepest-descent algorithm (30,000 steps), the systems were heated to 303.15 K over 200 ps in the NVT ensemble. Equilibration was performed in the NPT ensemble through six successive 20 ps stages with gradually decreasing positional restraints on heavy atoms, with the final stage unrestrained. Temperature and pressure were controlled using a Langevin thermostat with a collision frequency of 1.0 ps^-^¹ and Parrinello-Rahman barostat with a relaxation time of 2 ps (69, 70). Bonds involving hydrogen were constrained with LINCS (71). Production runs were carried out for 200 ns each with a 2-fs timestep.

### 4.4 Elastic normal mode analysis

Anisotropic network model (ANM) analysis was performed on full spike trimer structures using the ProDy software package to investigate local flexibility patterns and long-range allosteric communication pathways (72, 73). The analysis was conducted on refined crystal structures of wild-type Wuhan and engineered variants (Spike BA.2 NEC 1-4), focusing on the first chain of each trimer. The ANM was constructed using Cα atoms as network nodes with a cutoff distance of 15 Å for defining inter-residue contacts. The Hessian matrix was built based on harmonic spring interactions with uniform force constants between residues within the cutoff distance (74). Normal mode decomposition was performed through singular value decomposition to extract collective motions.

Local flexibility within the full spike context was quantified using the first 20 slowest normal modes. Squared fluctuations were calculated for each residue using ProDy’s calcSqFlucts function (75):

SeqFlucti=∑k=1N(uik)2λkwhere uik represents the eigenvector component for residue *i* in mode *k*, and *λ_k_* is the corresponding eigenvalue. RBD relative flexibility was computed as the mean squared fluctuation over RBD residues (319-541), providing a domain-averaged measure of conformational mobility within the trimeric spike context. This domain-averaged mean squared fluctuation, hereafter termed the *RBD relative MSF*, was computed within the context of the full trimeric Spike and is distinct from the per-residue RMSF derived from explicit-solvent molecular dynamics of the isolated, which reflects local domain dynamics outside the trimeric context.

Allosteric coupling between functional domains was quantified using ProDy’s calcPerturbResponse function (76, 77). The method calculates the linear response of each residue to unit perturbations applied elsewhere in the protein network. RBM→FP coupling strength was computed as the mean PRS response of fusion peptide residues (816-855) to perturbations at receptor-binding motif residues (438-506):


Coupling_RBM→FP = (1/|RBM|×|FP|) ∑∑ |PRS_ij|

where PRS_ij represents the response magnitude of residue j to perturbation of residue i. PRS matrices were normalized by the covariance diagonal to enable direct comparison across variants. All calculations were performed at 300 K.

### 4.5 Electrostatics potential surface

Electrostatic potentials for the modelled spike proteins, as previously described, were calculated by solving the linearized Poisson–Boltzmann (PB) equation using the Adaptive Poisson-Boltzmann Solver (APBS) software (78). Prior to electrostatic calculations, PDB2PQR (79) was used to prepare input structures by adding hydrogen atoms at pH 7.4, assigning protonation states based on pKa predictions with the PROPKA code (62), and generating PQR files with partial atomic charges and radii from the PARSE force field (80). The continuum electrostatic model treated the solvent as a high-dielectric medium (ϵ_solvent_ = 78.0) with a physiological ionic strength of 0.15 M monovalent salt, while the protein interior was modeled as a low-dielectric cavity (ϵ_protein_ = 4.0). The PB calculations were performed on a three-level focusing multigrid with grid spacings of approximately 1.0, 0.5, and 0.25 Å. The molecular surface was defined using a solvent probe radius of 1.4 Å, and the Stern ion exclusion layer was set to 2.0 Å. Temperature was set to 298.15 K for calculation of the thermal energy (k_B_T). Boundary conditions were set to a single Debye-Hückel sphere for all three focusing levels.

### 4.6 Study design and animals

This study evaluated the immunogenicity of four B-cell epitope–based pan-β-coronavirus mRNA vaccine candidates in a murine model. A total of 120 female BALB/c mice (63-69 days of age at arrival) were used. Animals were randomly assigned to eight experimental groups (n = 16 per group for Groups 1-7; n = 8 for Group 8). Animals were clinically healthy at study start and had not received any prior treatments that could interfere with the study objectives. All procedures were conducted by trained personnel, and animal health and welfare were monitored throughout the study.

Mice were assigned to the following groups:

- Group 1: Bivalent mRNA-LNP Comirnaty SARS-CoV-2 vaccine (prototype & Omicron BA.4/BA.5 strains)
- Group 2: EGFP mRNA-LNP (mock-vaccinated control)
- Group 3: Wuhan-based Spike mRNA-LNP
- Groups 4-7: Four engineered Spike pan-β-coronavirus mRNA-LNP vaccine candidates (Spike BA.2 NEC 1 to Spike BA.2 NEC 4)
- Group 8: Unvaccinated negative control (prime-boost cohort only)

Animals arrived at the facility 13 days prior to the first immunization. Upon arrival, mice were weighed, assigned to treatment groups, and individually identified by tattoo marking. Animals were acclimatized until Day -5, at which time body weights were recorded and pre-immune blood samples were collected to establish baseline seronegativity.

Two immunization regimens were evaluated: prime-boost regimen (vaccinations on Days 0 and 28), or prime-double-boost regimen (vaccinations on Days 0, 28, and 56). Vaccines were administered intramuscularly at a volume of 50 µL per dose. Injections were alternated between hind limbs (left on Day 0, right on Day 28, and left on Day 56). Test vaccines were dosed at 10 µg mRNA per animal (0.5 mg/kg assuming 20 g body weight), while the comparator vaccine (Comirnaty) was diluted to deliver 1 µg mRNA per animal (0.05 mg/kg). Comirnaty was administered at 1 µg per animal in its proprietary LNP formulation, reflecting the dose commonly used for this product in murine immunogenicity studies. All research-grade in-house constructs were administered at 10 µg using the eTheRNA LNP platform. Comirnaty was included as a clinical reference point rather than a dose- and formulation-matched experimental comparator; the full-length wild-type Wuhan Spike mRNA-LNP construct serves as the matched experimental comparator for design-related conclusions.

General health observations were performed daily throughout the study. Additional health checks were conducted on vaccination days to monitor for potential local or systemic adverse effects. Body weights were measured twice during acclimatization, on consecutive days surrounding each vaccination, and weekly thereafter as an indicator of general health and adequate food intake.

### 4.7 Blood collection and serum processing

Blood samples (~100 µL) were collected from the submandibular vein on Days -5, 35, and 63. Terminal blood samples were collected under general anesthesia during necropsy on Day 42 (prime-boost cohort) or Day 70 (prime-double-boost cohort) via orbital sinus blood draw. Blood samples were allowed to clot for at least 1 hour at ambient temperature and were centrifuged at 1,250 × g for 10 minutes. Sera from non-terminal collections were stored without aliquoting, whereas terminal sera were aliquoted to minimize freeze–thaw cycles. All serum samples were stored at -75 ± 10 °C until analysis.

### 4.8 Live virus neutralizing assay

Neutralizing antibody titers were determined using live virus neutralization assays against ancestral SARS-CoV-2 (Lelystad strain), Omicron BA.5, Omicron XBB.1.5, and SARS-CoV (HKU-39849) using terminal sera collected on Day 42. For the Pangolin-CoV GX and BtKY72 strains, the terminal sera collected from Day 70 was used. Heat-inactivated sera were serially diluted two-fold (starting dilution 1:10) in duplicate and incubated with 100 TCID_50_ of virus for 1.5 hours at ambient temperature before transfer to VERO-hSLAM cells in 96-well plates. Virus back-titration was performed for each assay to confirm the applied infectious dose. After two days of incubation, neutralization was assessed by immunoperoxidase monolayer assay (IPMA), visualizing viral Spike protein (SARS-CoV-2 variants and related strains) or Nucleoprotein (SARS-CoV). Staining was evaluated independently by two qualified observers. Neutralizing titers were defined as the reciprocal log_2_ value of the highest serum dilution that inhibited viral infection in at least 50% of wells. Non-neutralizing samples were assigned a value below the lower limit of detection.

### 4.9 SARS-CoV-2 spike binding antibody ELISA

Spike receptor-binding domain (RBD)-specific IgG antibodies against ancestral SARS-CoV-2, Omicron BA.5, and Omicron XBB.1.5 were quantified by indirect enzyme-linked immunosorbent assay (ELISA) using commercially available mouse anti–SARS-CoV-2 Spike/RBD IgG serology kits (ACROBiosystems, USA), according to the manufacturer’s protocols. Pre-immune sera (Day -5), terminal sera collected on Day 42, and non-terminal sera collected on Day 63 were analyzed. Serum samples were tested at predefined dilutions selected based on preliminary optimization experiments. Absorbance was measured at 450 nm with wavelength correction at 630 nm. Background-corrected optical density values were calculated by subtracting the signal obtained from blank wells containing dilution buffer only.

### 4.10 mRNA vaccine design, production and formulation

The mRNA vaccine constructs were designed and manufactured by eTheRNA immunotherapies NV using their proprietary mRNA vector platform. DNA templates encoding the full-length vaccine constructs were generated from plasmids and used for *in vitro* transcription. mRNA synthesis was performed using CleanCap AG technology to generate a co-transcriptionally capped 5′ end, combined with a co-transcriptional poly(A) tail. Modified nucleotides were incorporated by substituting uridine with N1-methyl-pseudouridine to enhance mRNA stability and translational efficiency. Following *in vitro* transcription, mRNA was purified according to eTheRNA’s established manufacturing protocols, including enzymatic digestion of template DNA and chromatographic purification steps to remove residual contaminants. Purified mRNA was formulated into lipid nanoparticles (LNPs) suitable for intramuscular administration using proprietary formulation processes optimized to preserve RNA integrity, ensure efficient encapsulation, and support *in vivo* expression. All steps of mRNA synthesis, purification, and LNP formulation were conducted under conditions designed to maintain mRNA quality, translational competence, and immunogenic performance, and quality control assessments were performed by the manufacturer prior to release for *in vivo* use.

### 4.11 Heatmap generation and normalization of ELISA data

For visualization and comparison across vaccination groups and time points, a heatmap was generated using antigen-specific normalization. For each antigen, a single dilution within the optimal dynamic range was selected based on assay performance and variability. Only measurements obtained at these predefined dilutions were included in the analysis. To account for antigen-specific background and dynamic range differences, responses were normalized relative to the unvaccinated control group measured at day 42. Day 42 was selected because the unvaccinated negative-control cohort was terminated at this time point and thus provided the seronegative reference sera; the same fixed reference was then applied to both the Day 42 and Day 63 samples so that the two time points remain directly comparable and real changes over time are not masked by a shifting baseline. As unvaccinated animals remain seronegative, which was confirmed at Day −5 and Day 42, this background is stable across the interval assessed. Specifically, for each antigen, the median OD value of the unvaccinated group at day 42 was used as a reference, and all samples (Day 42 and Day 63) were expressed as log_2_ fold-change relative to this reference value. Normalized values were assembled into a matrix with antigens as rows and individual mice as columns. Columns were annotated by vaccination group and sampling day. Heatmaps were generated using the *pheatmap* package in R, with hierarchical clustering disabled to preserve the predefined ordering of antigens and vaccination groups. Color scales were centered at zero to reflect relative increases or decreases in IgG binding compared to unvaccinated controls.

### 4.12 Correlation of RBD conformational dynamics with neutralization breadth

To assess whether conformational dynamics of the RBD were associated with neutralizing antibody responses, we correlated elastic normal mode analysis flexibility metrics with virus neutralization titers using a linear mixed-effects modeling framework. Neutralization titers against SARS-CoV-2 prototype, BA.5, XBB.1.5, SARS-CoV HKU39849, BtKY72, and pangolin CoV were log_2_-transformed prior to analysis.

To account for repeated measurements across vaccine constructs and virus strains, a linear mixed-effects model was fitted using restricted maximum likelihood estimation (REML), with log_2_ neutralization titers as the response variable, RBD relative MSF as a fixed effect, and random intercepts for vaccine construct and virus strain:


titer_log2 ~ rbd_relative_msf + (1 | group) + (1 | strain)

This modeling strategy allowed estimation of the global association between RBD flexibility and neutralization potency while controlling for between-group (vaccine design) and between-strain (virus variant/species) variability. Model fitting was performed using the *lme4* package in R. Fixed-effect estimates and 95% confidence intervals were extracted using *broom.mixed*. For visualization, strain-specific mean log_2_ neutralization titers were plotted against RBD MSF values for each vaccine construct, with linear trend lines provided as visual guides.

## Data Availability

The original contributions presented in the study are included in the article/**Supplementary Material**. Further inquiries can be directed to the corresponding author.
