# Supplementary material for: AI-guided epitope engineering of a SARS-CoV-2 spike antigen for broad sarbecovirus neutralization
Source: Front Immunol. 2026 Sep 18;17:1919664. doi: 10.3389/fimmu.2026.1919664 (PMC13631046; doi:10.3389/fimmu.2026.1919664)
Supplement: Supplementary file 1 [file Table1.docx]

Supplementary Material

# 1 Supplementary Figures

**Supplementary Figure S 1:** Root Mean Square Deviation (RMSD) of the Cα backbone over the course of MD simulations. Time series (left) and probability density distributions (right) of RMSD values for the wild-type (WT) RBD and each mutant variant (RBD1–RBD4). RMSD was calculated relative to the energy-minimized starting structure. Density profiles were estimated via kernel density estimation (KDE). Greater overlap between WT and mutant distributions indicates preservation of the overall conformational ensemble upon mutation.

**Supplementary Figure S 2:** Root Mean Square Fluctuation (RMSF) per residue from MD simulations. Per-residue Cα fluctuations are shown for the wild-type RBD (salmon) and each mutant variant (RBD1–RBD4) across the simulated trajectory. Shaded areas represent the RMSF profile to facilitate visual comparison of local flexibility changes. Regions of elevated RMSF relative to WT indicate mutation-induced increases in local backbone mobility.

**Supplementary Figure S 3**: Structural determinants of cross-neutralization breadth in engineered BA.2 Spike variants. (A) Allosteric coupling between the RBM and fusion peptide (FP). Bar graph showing perturbation response scanning (PRS)-derived coupling strength for wild-type Wuhan Spike and engineered BA.2 variants (Spike BA.2 NEC 1-4). (B-E) SASA profiles across the RBD for individual Spike variants. Per-residue SASA values derived from molecular dynamics simulations are plotted for Spike BA.2 NEC 1 (B), Spike BA.2 NEC 2 (C), Spike BA.2 NEC 3 (D), and Spike BA.2 NEC 4 (E). (F-J) Electrostatic surface potential maps of the RBD interaction interface. Three-dimensional representations are shown for Wuhan Spike (F) and Spike BA.2 NEC 1-4 (G-J). Electrostatic potentials were calculated by solving the linearized Poisson–Boltzmann equation and are displayed on the molecular surface.

Supplementary Figure S 4: Distribution of conformational B-cell epitope lenght across the candidate PPV pool. (A) Distribution of the full structural span of predicted conformational epitopes (probability >0.9), defined as the total number of residues encompassed by each PPV including both direct contact positions and non-contact positions that constitute the conformational architecture of the epitope. Full epitope spans range from 4 to 215 residues, reflecting the discontinuous, three-dimensional nature of conformational epitopes, which can involve residues distributed across non-contiguous regions of the primary sequence brought into spatial proximity by protein folding. (B) Distribution of the number of direct antibody-contact positions within each predicted conformational B-cell epitope, across all PPVs retained after probability filtering (threshold > 0.9).

**Supplementary Figure S 5:** RBD sequence alignment of WT BA.2 with Spike BA.2 NEC-1 to NEC-4, substituted residue highlighted in each
